# Occupational Practitioner’s Role in the Management of a Crisis: Lessons Learned from the Paris November 2015 Terrorist Attack

**DOI:** 10.3389/fpubh.2016.00203

**Published:** 2016-09-20

**Authors:** Alexis Descatha, Alice Huynh Tuong, Service Médical Du Raid, Pierre Coninx, Michel Baer, Thomas Loeb, Thomas Despréaux

**Affiliations:** ^1^AP-HP UVSQ, Occupational Health Unit, University Hospital of West Suburb of Paris, Garches, France; ^2^AP-HP, EMS (Samu92) University Hospital of West Suburb of Paris, Garches, France; ^3^UMR-S 1168, Versailles St-Quentin University UVSQ, Villejuif, France; ^4^U1168, INSERM, VIMA: Aging and Chronic Diseases. Epidemiological and Public Health Approaches, Villejuif, France; ^5^Police Intervention Unit RAID, Bièvres, France

**Keywords:** crisis, occupational, management, health practitioner, terrorism

## Abstract

In massive catastrophic events, occupational health practitioners are more and more frequently involved in the management of such situations. We aim to describe the multiple aspects of the role that occupational health practitioners might play, by focusing on the recent example of the Paris terrorist attack of November 2015. During and after the Paris attack, occupational practitioners, in collaboration with emergency and security professionals, were involved in psychological care, assembling information, follow-up, return-to-work, and improving in-company safety plans. Based on this experience and other industrial disasters, we distinguish three phases: the critical phase, the post-critical phase, and the anticipation phase. In the critical phase, the occupational practitioner cares for patients before the emergency professionals take charge, initiates the psychological management, and may also play an organizational role for company health aspects. In the post-critical phase, he or she would be involved in monitoring those affected by the events and participate in preventing, to the extent possible, posttraumatic stress disorder, helping victims in the return-to-work process, and improving procedures and organizing drills. In addition to their usual work of primary prevention, occupational practitioners should endeavor to improve preparedness in the anticipation phase, by taking part in contingency planning, training in first aid, and defining immediately applicable protocols. In conclusion, recent events have highlighted the essential role of occupational health services in anticipation of a crisis, management during the crisis, and follow-up.

## Introduction

In massive catastrophic events, emergency professionals deal with the caretaking of many victims. Occupational health services and practitioners are also being more and more frequently solicited.

These practitioners play an important role in enhancing preparedness for emergencies, responding to industrial disasters, pandemics, and other types of mass casualties, as well as in the follow-up of post-disaster emergency teams (1, 2). Companies can benefit from the skills and resources of occupational health practitioners, in managing acute situations, following workers, and even participating in prevention planning for such events (3–5).

In 2001, in the same month in which the US and the world had to deal with 9/11, France faced one of its worst industrial disasters near Toulouse [31 deaths, 2500 injured (6)]. We learned of the essential role of the occupational practitioner in such events and in terrorism preparedness, as already established in other countries (7).

Despite the differences in work and medical environments across the world, here, we aim to describe the multiple aspects of the role that occupational health practitioners can play in the management of exceptional situations, by focusing on the recent example of the Paris terrorist attack of November 2015.

## Focusing on the Paris Crisis

The Paris attacks happened on Friday, November 13, 2015. The first night, multiple shootings occurred at five different sites. At the Bataclan location, fatal shootings gave way to hostage taking. Three explosions occurred around the Stade de France; emergency teams supported over 60 victims and faced the challenge of overseeing an orderly evacuation of 72,000 spectators. In the night, these emergency teams had to face with 129 dead on sites and more than 300 injured. It ended with a final assault against terrorists on Wednesday, November 18.

Along with other health professionals, many occupational practitioners were involved in these catastrophic events (8).

They were solicited for many reasons: requests for information from managers about what to do in case of an attack (specific procedures, security measures, and first aid), specifics of the management and monitoring of victims, and of professionals and volunteers involved in their care. Most of these occupational health professionals were also involved with the care of indirect casualties of these attacks: people emotionally close to direct victims, people in whom the attacks reactivated earlier trauma, or simply compassionate people still requiring care.

After the crisis, the occupational health services played an active role in helping workers to continue to live and work “as usual,” improving the return-to-work process of direct and indirect victims, and handling the long-term follow-up of professionals and volunteers involved. Police, hospitals, and other public facilities, as well as private companies, improved their security plans with security and health services following government proposals and their rules on what to do in case of inner attack.

In order to anticipate future crises, most companies with sensitive sites intensified their work on prevention of such attacks. They have improved access to security, reinforcing video surveillance and preparedness for first aid. First, bystander actions (“secure, triage, damage control, evacuate”) have been rehearsed in drills organized by occupational health and security services: getting everyone to a safe and secure place, giving information to the police about the number of terrorists, and improving access to security centers, and videos for the police. Occupational health professionals have also trained first-aiders in the fundamentals of damage control and rapid evacuation.

## Generalization of the Role of the Occupational Practitioner in Managing a Crisis

Taking into account previous industrial disasters, biological pandemic alerts, and this experience, we can highlight three phases: the critical phase, the post-critical phase, and the anticipation phase.

### Critical Phase

If present at the scene, the physician would need to take care of patients by providing first aid and care in a secure zone before professional responders (emergency medical services – EMS, firemen, and police) arrive. A specifically trained occupational nurse could have a synergistic role, especially with preestablished protocols.

Initial psychological care is usually managed by the occupational physicians and nurses, in interaction with primary care, and with the emergency psychologist and psychiatrist.

Occupational practitioners would also have an organizational role on the disaster site: choosing how to secure the area in cooperation with professional responders, deciding whether or not decontamination is needed, according to the potential risks, trying to anticipate the needs of on-site emergency workers, from material (water, food, blankets, beds, oxygen, drugs, etc.) to human needs (organizing shifts, psychological help, etc.). They would also contribute to establishing an exhaustive list of sick or injured victims, to be used for the post-event monitoring of everyone.

Occupational practitioners are also internal health experts for company managers and would be involved in the management and monitoring of casualties as well as indirect victims. This expert role would provide a decisive link between professional responders, company executives, and administrative authorities. Beyond their technical expertise, the network of occupational professionals could prove to be a valuable asset in such crises (toxicologists, poison control centers, infectious diseases, psychological units, etc.).

### Postcrisis Phase

The post-critical phase can be defined as the period starting when all victims have been identified, managed, and sent for appropriate care, and continuing up to the resumption of normal activity by the company. This phase may last from hours to months. During this phase, the occupational practitioner would play a substantial role in monitoring people that were directly concerned by the events as well as screening workers who were indirectly involved. The occupational practitioner might be involved in diagnosing and handling patients with posttraumatic stress disorder. Screening should be considered for high-risk individuals. Even though psychological debriefing has no demonstrated benefit, there are benefits of early intervention and a proper follow-up of employees after a critical event by occupational health services (9–11). Return-to-work processes for these workers would have to be planned. Specific procedures for emergency care could also be improved, in cooperation with security, health, and safety services, for the handling of such events.

Drills are usually carried out to provide preparation for future emergencies in companies that have experienced such an event.

### Anticipation Phase

The most important role of the occupational practitioner should be implemented *before* the crisis, in order to prevent, anticipate, and limit its effects. In addition to their usual work of primary prevention, occupational practitioners should endeavor to improve preparedness by taking part in contingency planning, conducting extensive staff training in first aid, and defining immediately applicable protocols that vary according to the kind of event (biological or chemical hazard, accident, or terrorism).

## Conclusion

Recent events have highlighted the essential role of occupational health services in the anticipation of a crisis, management during the crisis, and follow-up.

## Author Contributions

All the authors have participated in the conception and design of the study, data acquisition, analysis, and interpretation, drafting the article or revising it critically for important intellectual content. They have all approved the final version submitted.

## Conflict of Interest Statement

No relevant conflicts of interest in accordance with ICMJE. The authors declare that the research was conducted in the absence of any commercial or financial relationships that could be construed as a potential conflict of interest. The authors are paid for their respective affiliations. In addition, AD has received money for his editing work (Editor-in-chief of Archives des Maladies Professionnelles) from Elsevier-Masson. AD and MB are members of ICOH.
